# RMBase v3.0: decode the landscape, mechanisms and functions of RNA modifications

**DOI:** 10.1093/nar/gkad1070

**Published:** 2023-11-13

**Authors:** Jiajia Xuan, Lifan Chen, Zhirong Chen, Junjie Pang, Junhong Huang, Jinran Lin, Lingling Zheng, Bin Li, Lianghu Qu, Jianhua Yang

**Affiliations:** MOE Key Laboratory of Gene Function and Regulation, State Key Laboratory of Biocontrol, School of Life Sciences, Sun Yat-sen University, Guangzhou 510275, China; MOE Key Laboratory of Tumor Molecular Biology and Key Laboratory of Functional Protein Research of Guangdong Higher Education Institutes, Institute of Life and Health Engineering, College of Life Science and Technology, Jinan University, Guangzhou 510632, China; MOE Key Laboratory of Gene Function and Regulation, State Key Laboratory of Biocontrol, School of Life Sciences, Sun Yat-sen University, Guangzhou 510275, China; MOE Key Laboratory of Gene Function and Regulation, State Key Laboratory of Biocontrol, School of Life Sciences, Sun Yat-sen University, Guangzhou 510275, China; MOE Key Laboratory of Gene Function and Regulation, State Key Laboratory of Biocontrol, School of Life Sciences, Sun Yat-sen University, Guangzhou 510275, China; MOE Key Laboratory of Gene Function and Regulation, State Key Laboratory of Biocontrol, School of Life Sciences, Sun Yat-sen University, Guangzhou 510275, China; MOE Key Laboratory of Gene Function and Regulation, State Key Laboratory of Biocontrol, School of Life Sciences, Sun Yat-sen University, Guangzhou 510275, China; Human Phenome Institute, Fudan University, 825 Zhangheng Road, Shanghai 201203, China; MOE Key Laboratory of Gene Function and Regulation, State Key Laboratory of Biocontrol, School of Life Sciences, Sun Yat-sen University, Guangzhou 510275, China; MOE Key Laboratory of Gene Function and Regulation, State Key Laboratory of Biocontrol, School of Life Sciences, Sun Yat-sen University, Guangzhou 510275, China; MOE Key Laboratory of Gene Function and Regulation, State Key Laboratory of Biocontrol, School of Life Sciences, Sun Yat-sen University, Guangzhou 510275, China; MOE Key Laboratory of Gene Function and Regulation, State Key Laboratory of Biocontrol, School of Life Sciences, Sun Yat-sen University, Guangzhou 510275, China

## Abstract

Although over 170 chemical modifications have been identified, their prevalence, mechanism and function remain largely unknown. To enable integrated analysis of diverse RNA modification profiles, we have developed RMBase v3.0 (http://bioinformaticsscience.cn/rmbase/), a comprehensive platform consisting of eight modules. These modules facilitate the exploration of transcriptome-wide landscape, biogenesis, interactome and functions of RNA modifications. By mining thousands of epitranscriptome datasets with novel pipelines, the ‘RNA Modifications’ module reveals the map of 73 RNA modifications of 62 species. the ‘Genes’ module allows to retrieve RNA modification profiles and clusters by gene and transcript. The ‘Mechanisms’ module explores 23 382 enzyme-catalyzed or snoRNA-guided modified sites to elucidate their biogenesis mechanisms. The ‘Co-localization’ module systematically formulates potential correlations between 14 histone modifications and 6 RNA modifications in various cell-lines. The ‘RMP’ module investigates the differential expression profiles of 146 RNA-modifying proteins (RMPs) in 18 types of cancers. The ‘Interactome’ integrates the interactional relationships between 73 RNA modifications with RBP binding events, miRNA targets and SNPs. The ‘Motif’ illuminates the enriched motifs for 11 types of RNA modifications identified from epitranscriptome datasets. The ‘Tools’ introduces a novel web-based ‘modGeneTool’ for annotating modifications. Overall, RMBase v3.0 provides various resources and tools for studying RNA modifications.

## Graphical Abstract

**Graphical Abstract:**
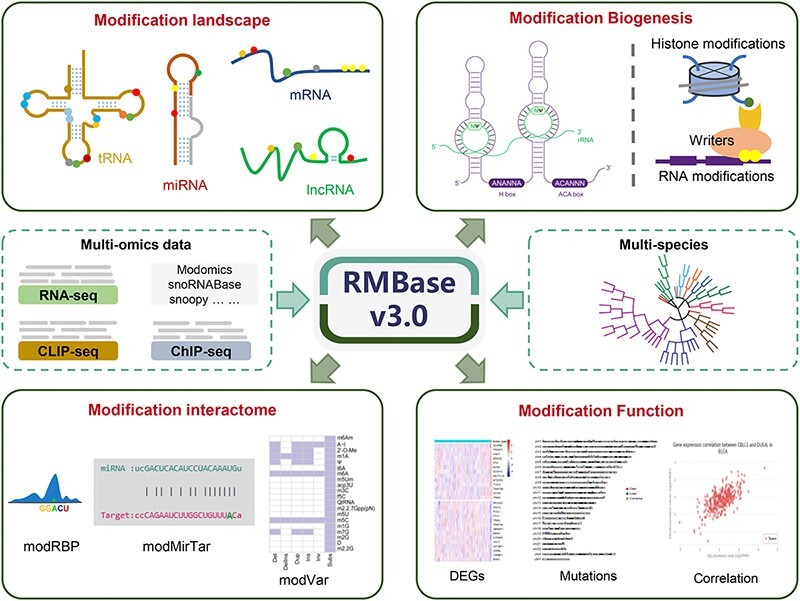

## Introduction

Epitranscriptomics, or RNA epigenetics, refers to the presence of chemical modifications on RNAs post-transcriptionally (1). Over 170 chemical modifications have been confirmed on various RNA types, such as rRNAs, tRNAs, mRNAs and lncRNAs. Moreover, these modifications are widespread exist in eukaryotes, bacteria, and archaea (2). Recent research has primarily focused on RNA modifications with high intracellular abundance, such as *N*^6^-methyladenosine (m^6^A), *N*^1^-methyladenosine (m^1^A), *N*^5^-methylcytosine (m^5^C), *N*^7^-methylguanosine (m^7^G), pseudouridine (Ψ), 2′-*O*-methylation (2′-*O*-Me or Nm), *N*^4^‐acetylcytidine (ac^4^C) and adenosine-to-inosine (A-I). The installation of these modifications is dynamically regulated by enzymes or guided by snoRNAs (1). RNA modification is endowed with diverse regulatory functions in RNA processing, transport, translation and degradation (3–5). This intricate orchestration is achieved through the regulation of RNA modifications by RNA-modifying proteins (RMPs), ultimately influencing the outcome of gene expression and a myriad of cellular processes (3–5). Notably, aberrant expression of RMPs can perturb normal cellular processes and contribute to the development of human diseases. However, the prevalence, biological roles and functional mechanisms of the majority of RNA modifications remain unclear.

Recent advances in high-throughput RNA modification technologies have produced tremendous amounts of epitranscriptome sequencing data, and enable the detection and profiling of known and novel RNA modification sites at unprecedented sensitivity and depth (6). While there are several pipelines available for downstream analysis, such as identifying RNA modification sites and elucidating their functions (3–6), their specificity for only one type of modification significantly constrains their broad applicability. Therefore, there remain several challenges for analyzing the epitranscriptome sequencing data. Firstly, the absence of a unified pipelines hinders the effective mining of large-scale high-throughput sequencing data for the precise detection of RNA modifications. Secondly, there is a great need for reliable and universally applicable computational methods to investigate the biogenesis mechanisms and biological roles of the diverse array of RNA modifications. Lastly, a comprehensive platform is required to analyze global transcriptome-wide RNA modifications, along with their distinctive characteristics across various species. Addressing above-mentioned challenges is crucial for uncovering the prevalence, biogenesis mechanisms and functions of various RNA modifications.

**Figure 1.**
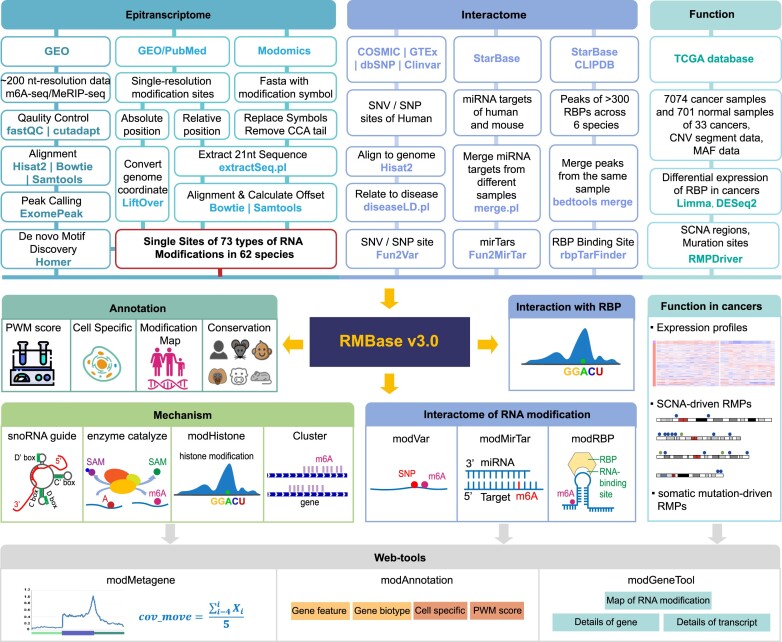
The workflow of RMBase v3.0. RMBase v3.0 serves as a comprehensive resource for deciphering RNA modification maps, biogenesis mechanisms, functions, interactomes, evolutionary conservation, and disease variations. It explores RNA modification landscapes across 62 species by *de novo* identifying m^6^A sites from ∼200 nt resolution high-throughput sequencing data and integrating modifications with single nucleotide precision from Modomics and publications (top left). RMBase v3.0 assigns scores to each RNA modification site using PWMs, enriches annotations with genomic information, cell lines and experimental datasets, and unveils their evolutionary conservation in different mammals. The platform investigates RNA modification biogenesis mechanisms at both genome and transcriptome levels (middle left). It delves into the interactome of RNA modifications with RBPs, miRNA targets, SNPs, and SNVs, while elucidating the biological roles of RMPs in tumors through analyzing RNA-seq from normal and tumor samples (top right and middle right). Based on the above-mentioned data, RMBase v3.0 also provides three web-based tools (bottom) for users to perform customized analyses.

Here, we presented RMBase v3.0 (http://bioinformaticsscience.cn/rmbase/), the most comprehensive platform designed to decipher the transcriptome-wide landscapes, biogenesis, interactome and functional roles of RNA modifications (Figure 1). By leveraging novel computational pipelines to mine thousands of epitranscriptome sequencing datasets, RMBase v3.0 reveals transcriptome-wide map of 73 RNA modifications for 62 species spanning mammals, plants, vertebrates, fungi, insects, metazoa, protists, bacteria and viruses (Table 1). Notably, RMBase v3.0 illustrates the biogenesis mechanisms of several types of RNA modifications that are catalyzed by enzymes or guided by snoRNAs. RMBase v3.0 has provided a systematic exploration for the potential co-localization of RNA modifications with histone modifications by analyzing 517 ChIP-seq datasets. Importantly, by integrating this data with thousands of RNA-seq datasets, we explored the dysregulated expression profiles and mutation maps of 146 RMPs and unveiled their potential roles in 18 types of cancers. Additionally, RMBase v3.0 illuminated the relationships between 73 RNA modifications and various interacting factors and the enriched motifs for 11 types of RNA modifications. RMBase v3.0 also offers a comprehensive collection of webpages and graphic visualizations to perform further analyses for the underlying mechanisms and functions of RNA modifications.

**Table 1.** RNA modification statistics across 62 species in RMBase v3.0

**Species**	**m^6^A**	**m^1^A**	**m^5^C**	**Ψ**	**2**′**-O-Me**	**m^7^G**	**A-I**	**ac4C**	**Others**
*Homo sapiens*	234464	1162	46025	15078	8490	919	144122	1861	1484
*Mus musculus*	220022	91	47839	22518	1193	55	8864	0	950
*Macaca mulatta*	16699	0	0	0	0	0	0	0	0
*Pan troglodytes*	27828	0	0	0	0	0	0	0	0
*Rattus norvegicus*	43880	75	118	1317	3418	46	10	24	977
*Sus scrofa*	62116	0	0	0	0	0	0	0	0
*Mesocricetus auratus*	0	2	0	5	0	0	0	0	9
*Oryctolagus cuniculus*	0	88	98	225	46	70	0	0	493
*Ovis aries*	0	24	35	35	0	15	0	0	132
*Bos taurus*	0	127	94	381	105	29	0	16	770
*Arabidopsis thaliana*	35373	0	0	76	286	0	0	0	0
*Brassica napus*	0	49	0	175	128	59	0	0	425
*Chlamydomonas reinhardtii*	0	0	2	30	34	0	0	0	9
*Cucumis sativus*	0	12	14	34	0	0	0	0	105
*Glycine max*	0	0	0	4	1	1	0	0	7
*Nicotiana tabacum*	0	25	0	134	123	0	0	0	296
*Phaseolus vulgaris*	0	35	19	78	42	15	0	12	256
*Solanum tuberosum*	0	18	14	46	17	0	0	16	117
*Spinacia oleracea*	0	11	0	89	81	17	0	0	251
*Zea mays*	0	0	0	15	9	0	0	0	33
*Bombyx mori*	0	82	178	185	95	33	0	0	453
*Caenorhabditis elegans*	0	0	0	296	151	0	18	0	106
*Xenopus laevis*	0	55	86	104	46	35	0	0	270
*Danio rerio*	5071	0	0	5	40	0	0	0	0
*Gallus gallus*	0	4	0	36	55	2	0	0	37
*Candida albicans*	0	1	1	3	1	0	0	1	6
*Fusarium graminearum*	0	0	0	0	0	0	48493	0	0
*Fusarium verticillioides*	0	0	0	0	0	0	5227	0	0
*Neurospora crassa*	0	22	14	36	0	10	47345	0	134
*Pichia jadinii*	0	40	43	108	31	17	0	7	311
*Saccharomyces cerevisiae*	24486	151	231	2147	263	75	66	120	1767
*Schizosaccharomyces pombe*	1	8	8	41	18	9	0	0	87
*Drosophila melanogaster*	56	78	46	614	559	47	5049	13	494
*Tetrahymena thermophila*	0	50	49	165	81	0	0	0	205
*Bacillus subtilis*	15	6	0	99	3	39	0	0	185
*Clostridium acetobutylicum*	0	0	0	0	1	1	0	0	0
*Escherichia coli*	243	0	21	206	55	66	4	2	508
*Geobacillus stearothermophilus*	1	3	0	11	5	3	0	0	25
*Halobacterium salinarum*	0	0	10	17	10	0	0	0	31
*Halococcus morrhuae*	0	0	0	1	0	0	0	0	1
*Haloferax volcanii*	0	0	51	79	51	0	0	5	167
*Lactococcus lactis*	11	2	0	0	0	30	0	1	64
*Mycobacterium smegmatis*	0	1	0	1	0	1	0	0	1
*Mycoplasma capricolum*	12	10	0	38	2	17	0	0	78
*Mycoplasma mycoides*	4	1	0	4	0	3	0	0	14
*Pseudomonas aeruginosa*	1560	0	0	0	0	0	0	0	0
*Pyrococcus abyssi*	0	0	0	0	0	0	0	0	1
*Rhodospirillum rubrum*	0	0	0	2	0	1	0	0	4
*Salmonella typhimurium*	0	0	0	19	7	3	0	0	42
*Spiroplasma citri*	0	0	0	3	0	1	0	0	3
*Streptomyces coelicolor A3 (2)*	0	0	0	1	0	0	0	0	1
*Streptomyces griseus*	0	26	0	4	25	18	0	0	48
*Sulfolobus acidocaldarius*	0	1	1	0	4	0	0	0	1
*Sulfolobus solfataricus*	0	0	0	0	0	0	0	0	24
*Synechococcus elongatus PCC 6301*	0	0	0	8	1	1	0	0	11
*Synechococcus* sp.*PCC 7002*	0	0	0	2	1	1	0	0	5
*Synechocystis* sp.	0	0	0	1	0	0	0	0	3
*Thermoplasma acidophilum*	0	4	0	10	4	2	0	0	15
*Thermotoga maritima*	0	0	1	0	1	1	0	0	5
*Thermus thermophilus*	0	4	12	19	17	6	0	0	28
*Enterobacteria phage T4*	0	0	0	14	6	3	0	0	39
*Enterobacteria phage T5*	1	0	0	9	1	2	0	0	10

The table lists the number of sites for each RNA modification type in 62 species. m^6^A is *N*^6^-methyladenosine, m^1^A is *N*^1^-methyladenosine, m^5^C is 5-methylcytosine, Ψ is pseudouridylation, 2′-*O*-Me is 2′-*O*-methylation, m^7^G is *N*^7^-methylguanosine, A-I is a type of RNA editing, ac^4^C is *N*^4^-acetylcytidine, and ‘Others’ contains other RNA modification types.

## Materials and methods

### Collection of RNA modifications datasets

We collected and integrated thousands of RNA modification sequencing datasets from 62 different species (Supplementary Table S1). High-throughput sequencing datasets for RNA modifications were downloaded from Gene Expression Omnibus (GEO) (7). Among them, m^6^A high-throughput datasets are classified into two categories according to the mapping resolution: techniques with single nucleotide resolution like miCLIP (8), m6A-CLIP/IP (9), MAZTER-seq (10), DART-seq (11) and m6A-REF-seq (12), and techniques with limited resolution like m6A-seq and MeRIP-seq (13). m^1^A high-throughput datasets include m1A-seq (14) and m1A-MAP (15). m^5^C high-throughput datasets include m5C-RIP and Bisulfite-seq (13). m^7^G high-throughput datasets include m7G-seq (16), BoRed-seq and m7G-RIP-seq (17). Ψ high-throughput datasets consist of Ψ-seq (18), CeU-seq (19), Pseudo-seq (20) and PSI-seq (21), BID-seq (22) and PRAISE (23). 2′-*O*-Me high-throughput datasets include Nm-seq, RiboMeth-seq, RibOxi-seq and 2OMe-seq (24). ac^4^C high-throughput datasets include ac4C-seq (25) and acRIP-seq (26). A-I datasets were curated from various public databases, including FairBase (27), REDIportal (28), RADAR (29) and DARNED (30). Additionally, we integrated experimentally identified modifications derived from over 100 studies and 5 public databases including MODOMICS (31), snOPY (32), Yeast-snoRNADataBase (33), snoRNABase (34) and NCBI PubMed (35).

### Genome sequences and annotations of 62 species

For identification and functional analysis of RNA modification, we collected genome sequences and gene annotations for all 62 species that were downloaded from GENCODE (36) project for human and mouse, NCBI (37) and Ensembl (38) for other species (Supplementary Table S2). For miRNAs, tRNAs and repetitive sequences, we downloaded the annotations from miRbase (39), GtRNAdb (40) and UCSC genome browser (41) respectively. To ensure consistency and accuracy in downstream analysis, we standardized the annotations from different sources into the same version and file format for each species. Furthermore, we divided genes into six biotypes: mRNA, lncRNA, sncRNA, pseudogene, repeat elements, and intergenic, based on the criteria defined in GENCODE and Ensembl. Additionally, we further categorized genes into five features: 5′-UTR, 3′-UTR, CDS, exon and intron as required for the analysis.

### Identifying and annotating RNA modifications

For m^6^A high-throughput sequencing data, adaptor sequences were removed with cutadapt v1.11 (42) (parameters: -m 20 -q 20) and the trimmed sequences were aligned to corresponding genomes with HISAT2 (43) (–no-softclip –no-unal). m^6^A modification peaks were called by exomePeak (44) with the strict criteria (FDR < 0.05, FC ≥ 2 and *P*-value < 0.01) and m^6^A modification sites were determined by searching for consensus RRACH motifs (R denotes A or G, H denotes A, C or U) within the peaks. We scored each m^6^A site with position weight matrices (PWMs) and annotated them with cell type, data type, experiment datasets (support experimental number ≥ 2 and PWM score ≥ 3). For RNA modifications obtained from other databases like MODOMICS, the modified bases within the sequences were replaced with the reference bases, and the new sequences were then aligned to the corresponding genomes. RNA modifications derived from publications were standardized to match the versions of the respective genomes. For RNA modifications that were characterized by their relative positions to specific genes particularly in rRNAs and tRNAs, modified sequences that were extended by an additional 10 nt in both 5′- and 3′-directions centered on the RNA modification site were aligned to the corresponding genomes. Subsequently, the offsets were calculated to determine the genomic coordinates of RNA modification sites in different copies of the same rRNA or tRNA. All of the aforementioned RNA modifications were integrated and annotated with the prepared gene annotations. The evolutionary conservation of mammalian m^6^A modifications was determined by liftOver or phyloP (41). RNAfold (45) was employed to analyze the potential secondary structures within 15 nt upstream and downstream of RNA modification sites.

All the annotated RNA modification sites were systematically analyzed to identify RNA modification clusters in the transcriptome. The distribution and abundance of RNA modifications were examined at the level of individual genes. To ensure accuracy, only RNA modification sites supported by a minimum of two datasets (support experimental number ≥ 2) were considered as candidates for cluster formation. RNA modification cluster were defined using the following criteria: (i) there were at least 10 RNA modification sites within a cluster; (ii) the distance between each RNA modification site within the cluster should be ≤100 nt; (iii) the average distance between all the RNA modification sites within the cluster should be less than or equal to 50 nt.

### Detecting RNA modifications guided by the snoRNAs

For the Ψ modifications guided by snoRNAs, we firstly curated a snoRNA fasta file that include 639 H/ACA snoRNA sequences from snoDB (46) and 2718 Ψ-modified-sequences that were extended by an additional 10nt in both 5′- and 3′-directions centered on the Ψs stored in RMBase v3.0. We predicted the base-pairs between H/ACA snoRNAs and Ψ-modified-sequences using snoSeeker software with score >10 (47). In a parallel effort, we employed the same software to predict base-pair interactions between 1326 C/D snoRNA sequences and 4627 Nm-modified sequences (47).

### Exploring the association between histone modifications and RNA modifications

To systematically investigate the potential association of histone modifications with RNA modifications, we gathered 527 ChIP-seq datasets for 14 types of histone modifications in 12 different cell lines from ENCODE (48) and all RNA modifications stored in RMBase v3.0. We characterized the distribution abundance of histone modifications of 1000 bp upstream and downstream of RNA modification sites and fitted it using polynomial regressive rule. A type of histone modification was considered as candidate to associate RNA modifications if the following criteria are met: (i) the *F* test *P*-value of regressive equation is lower than 0.01; (ii) the adjusted *R*-squared is at least 0.8; (iii) the distance of the abundant summit from RNA modification sites is ≤15. Pearson correlation coefficient analysis was performed on the abundance of RNA modifications and histone modifications within the genome. We applied this method to explore the co-localization between histone modifications with various RNA modifications, including m^6^A, m^1^A, m^5^C, m^7^G, Ψ and 2′-*O*-Me. RMBase v3.0 contains 14 types of histone modifications, including H2AFZ, H3F3A, H3K27ac, H3K27me3, H3K36me3, H3K4me1, H3K4me2, H3K4me3, H3K79me2, H3K9ac, H3K9me1, H3K9me2, H3K9me3 and H4K20me1.

### Deciphering the expression profiles and mutation maps of RMPs in tumors

To explore the expression profiles of RMPs in various types of cancers, we gathered a collection of 146 human RMPs that have been reported in publications, as well as downloaded expression datasets of 33 tumors from The Cancer Genome Atlas (TCGA), of RECA from International Cancer Genome Consortium (ICGC) (49) and of AML from GEO platform (GSE48846 and GSE49642). The ‘voom’ algorithm from the limma package (50) and DESeq2 package (51) were used to perform differential expression profiles of RMPs. Specifically, we focused on 18 types of tumors that contain at least 10 tumor samples and 10 normal samples available, and obtained the overlapped differential expressed genes (DEGs) between DESeq2 and limma results with a fold change (FC) ≥ 1.5 and a *P*-value <0.05. The Pearson correlation coefficient and *P*-value were used to assess the correlation, including positive correlation (cor ≥ 0.5 and *P* < 0.05) and negative correlation (cor ≤ −0.5 and *P* < 0.05) between gene expression patterns in diverse samples (‘Correlation’ section of the ‘RMP’ module).

To investigate the mutation patterns of RMPs, we collected copy number variation (CNV) segment data and somatic mutation annotation format (MAF) files for the 16 types of tumors from TCGA. The significant SCNA regions were identified using GISTIC2 in TCGA (52). Potential tumor driver genes were detected by the DOTS-Finder(53) software in 16 types of tumors. Genes with false discovery rate (*q* value) ≤ 0.1 were considered as candidate driver oncogenes (OG) or tumor suppressor genes (TSG).

### Implementation of RMBase v3.0 web interfaces

All processed data and pipelines were stored in RMBase v3.0, a comprehensive and flexible platform that were constructed using HTML5, PHP7, CSS3 and Javascript. We employed several external packages and software to display the data stored in the RMBase v3.0, including Bootstrap v4.4.1 framework for organizing of web interfaces, MySQL for backend storage and query of processed data, the DataTables for presenting tabular results, HighCharts for visualizing diverse results, CGI, R and Perl for data analysis in the web-server.

## Database content and web interface

### Uncovering the RNA modification map across 62 species with the ModFinder pipeline

To comprehensively decipher the global map of RNA modifications, we developed a unified computational pipeline ‘ModFinder’ (Figure 1). Specifically, we analyzed 1880 epitranscriptome sequencing datasets to detect precise m^6^A modification sites, and constructed the alignments and distribution features of RNA modifications derived from public databases (detail information in Materials and methods). After rigorous quality control and filtering, we identified and obtained over one million accurate RNA modification sites for 73 types of RNA modifications across 62 different species. This includes 671 843 sites for m^6^A, 2268 sites for m^1^A, 95 010 sites for m^5^C, 1653 sites for m^7^G, 44 528 sites for Ψ, 15 507 sites for 2′-*O*-Me, 259 198 sites for A-I, 2078 sites for ac^4^C and 11 498 sites for other types (Table 1).

We further described the identified modification sites with comprehensive annotations, including gene names, gene types, biotype features, PWM scores, sequence context, RNA secondary structures, biogenesis mechanisms and cell sources. The global map of all RNA modifications in the transcriptome revealed a wide range of preferences for different RNA modifications (Supplementary Figure S1A). For instance, m^6^A modifications are most abundant in mRNA when compared to other RNA species (54), while tRNA is the most heavily modified RNA species in terms of abundant, density, and diversity of RNA modifications (3). Moreover, metagene plots for different RNA modifications generated using the ‘modMetagene’ provided by RMBase v3.0 showcased distinct distribution patterns, consistent with previous reports (55). For example, m^6^A is significantly enriched near the stop codon, a conservation observed across mammals, plants, yeast, zebrafish and so on (Supplementary Figure S2A). A-I tends to be predominantly situated in the 3′ UTR region in human and mouse, while it shows enrichment near the stop codon in fungi like *Neurospora crassa* and *Fusarium verticillioides* (Supplementary Figure S2B). Ψ is primarily localized in the CDS and 3′ UTR regions, and this conservation is observed in human, mouse, and yeast (Supplementary Figure S2C). m^5^C exhibits a widespread distribution in the CDS and 3′ UTR regions of human and mouse, with significant enrichment near the start codon (Supplementary Figure S2D). These results suggest the distinct patterns of different RNA modifications observed in various RNA are likely closely linked to their diverse biogenesis mechanisms and biological functions.

### Identification of clusters for various RNA modifications

Given that the clustered m^6^A modifications within genes serve a pivotal role in biological processes (56), we conducted a comprehensive analysis of the distribution patterns of RNA modifications to investigate the clusters of m^6^A, m^5^C, 2′-O-Me, Ψ and A-I in human transcriptome. We revealed a total of 5871 m^6^A clusters, 98 m^5^C clusters, 18 2′-O-Me clusters, 17 Ψ clusters, and 59 A-I clusters (Supplementary Figure S1B, Supplementary Table S3). This suggests that m^6^A, as previously reported (54), is frequently clustered, whereas the presence of only a few clusters in 2′-O-Me and Ψ appears to be more random. Furthermore, m^6^A clusters were observed to be distributed in mRNA, lncRNA and pseudogenes, with the majority (89.66%, 5264/5871) of them located in mRNA (Supplementary Figure S1C). Further statistical analysis revealed that a single mRNA can contain one or multiple m^6^A clusters, with length ranging from 120 nt to 3000 nt. Additionally, the average distance between m^6^A modification sites within these clusters is less than 50 nucleotides. These RNA modification clusters have been integrated into ‘modGene’ and annotated with Gene Ontology (GO) terms (57), providing users with a convenient way to explore their biological functions.

### RNA modifications guided by snoRNAs in diverse RNA species

**Figure 2.**
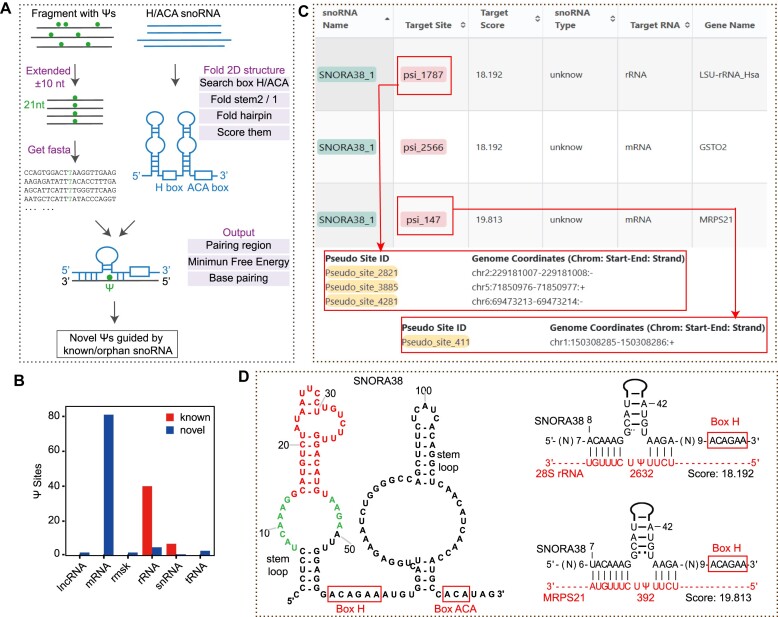
Identification of Ψ modification sites guided by snoRNAs using sno2Psi. (**A**) Flowchart depicts the workflow of sno2Psi. (**B**) the barplot shows that the distribution of known (red) and novel (blue) snoRNA-guided Ψs on different gene biotypes. (**C**) The screenshot from the ‘Browser’ of the ‘Mechanism’ module shows the SNORA38-guided Ψs and their genomic coordinate on the rRNA and mRNA. (**D**) The predicted 2D structure of SNORA38 has two stem loops and H/ACA boxes (left); The predicted base-pairings between SNORA38 (black) with 28S rRNA and MRPS21 mRNA (red) (right). Ψs in red indicated the putative Ψ site at 2632th U in 28S rRNA and 394th U in MRPS21 mRNA in human.

Eukaryotic Ψ and 2′-*O*-Me modifications are primarily catalyzed by H/ACA snoRNAs and C/D snoRNAs (58,59). To systematically identify snoRNA-guided Ψs installed on all various RNA molecules, we developed a novel pipeline called ‘Sno2Psi’ that incorporates our snoSeeker software (47) (Figure 2A, Materials and methods). Notably, we detected 141 human snoRNA-guided Ψs, including 94 novel snoRNA-guided Ψs and 47 known snoRNA-guided Ψs that located within rRNAs, tRNAs, mRNAs, lncRNAs and snRNAs (Figure 2B, Supplementary Table S4). Moreover, these Ψs were guided by 98 snoRNAs, including 82 known snoRNAs and 16 orphan snoRNAs that lack apparent complementarity to any known RNA targets (60) (‘Browser’ section of the ‘Mechanism’ module). For example, our results demonstrated new targeting relationships between orphan SNORA38 with 2 Ψ sites at 2632th (Ψ2632) uracil (U) of 28S rRNA and 392th U (Ψ392) of MRPS21 mRNA, suggesting that orphan SNORA38 potentially guide the formation of these 2 sites (Figure 2C, D, Supplementary Table S2).

We further developed another pipeline called ‘Sno2Nm’ to systematically detect C/D snoRNA-guided 2′-*O*-Me sites on various human RNA molecules (Supplementary Figure S3A). We identified 444 snoRNA-guided 2′-*O*-Me sites that located within rRNAs, tRNAs, lncRNAs, mRNAs, snRNAs and pseudogenes (Supplementary Figure S3B, Supplementary Table S5). These 2′-*O*-Me sites are guided by 144 known guide C/D snoRNAs and 29 orphan C/D snoRNAs. Overall, these results provide valuable mechanistic insights into the biosynthesis of Ψ and 2′-*O*-Me modifications.

### Investigation of co-localization between histone modifications and RNA modifications

**Figure 3.**
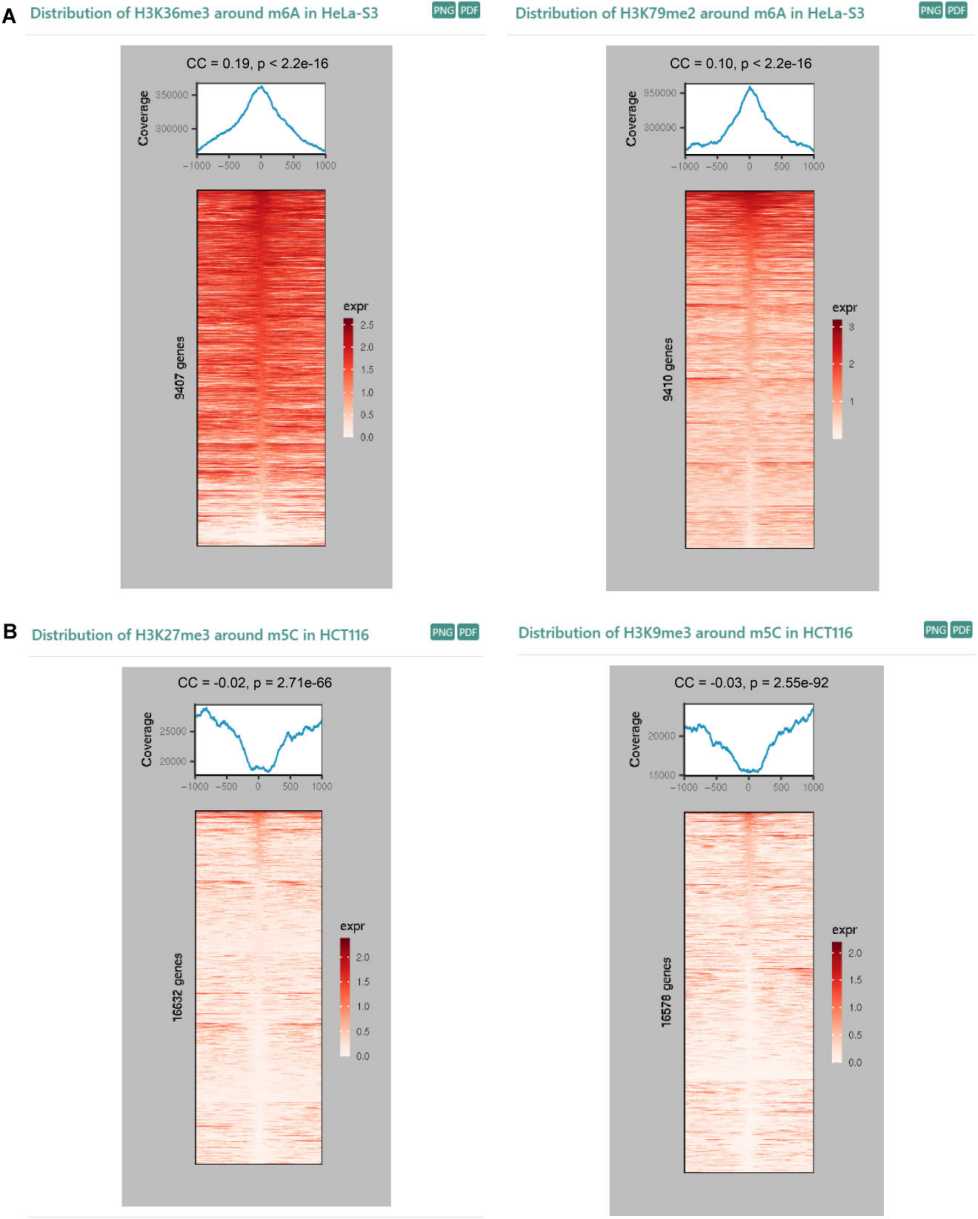
Distribution correlation of histone modification and RNA modification. (**A**) Distribution patterns of H3K36me3 and H3K79me2 modifications around m^6^A sites in RMBase v3.0 in HeLa cell line. (**B**) Distribution patterns of H3K27me3 and H3K9me3 modifications around m^5^C sites in RMBase v3.0 in HCT116 cell line. Pearson correlation coefficient (CC) and *P*-value are used to evaluate the significance of the potential correlation between RNA modifications and histone modifications. The m^6^A peaks used for Pearson correlation analysis was downloaded from GEO (GSE76414), while the m^5^C peaks were generated by extending 20 nt upstream and downstream from m^5^C sites that stored in RMBase v3.0.

we developed the ‘modHistone’ pipeline based on the polynomial regression to perform the integrated analysis of ChIP-seq data for 14 types of histone modifications and epitranscriptome sequencing data for m^1^A, m^6^A, m^5^C, m^7^G, Ψ, and 2′-*O*-Me across 12 different cell lines (Materials and methods). We inferred the co-localization relationship between histone modifications and RNA modifications by investigating the distribution patterns of the histone modifications near RNA modification sites and performing the Pearson correlation analysis on their respective abundances across the genome. Specifically, a positive correlation is observed when there is a significant enrichment peak of histone modifications at RNA modification sites, while a negative correlation is identified when there is a clear valley in the distribution of histone modifications at RNA modification sites. The Pearson correlation coefficient is used to further assess the significance of the potential correlation between the two. For instance, the distribution patterns revealed a strong overlap between the chromosome positions of m^6^A and H3K79me2, consistent with the well-characterized relationship seen with H3K36me3 (61), despite they both showed a weak positive correlation (CC < 0.2) (Figure 3A). However, m^5^C exhibits a significant negative correlation with H3K27me3 and H3K9me3 (Figure 3B). In addition, the results obtained from this pipeline have been integrated into the ‘Co-localization (modHistone)’ module for users to browse.

### Exploration of expression profiles and mutation maps of RMPs in tumors

**Figure 4.**
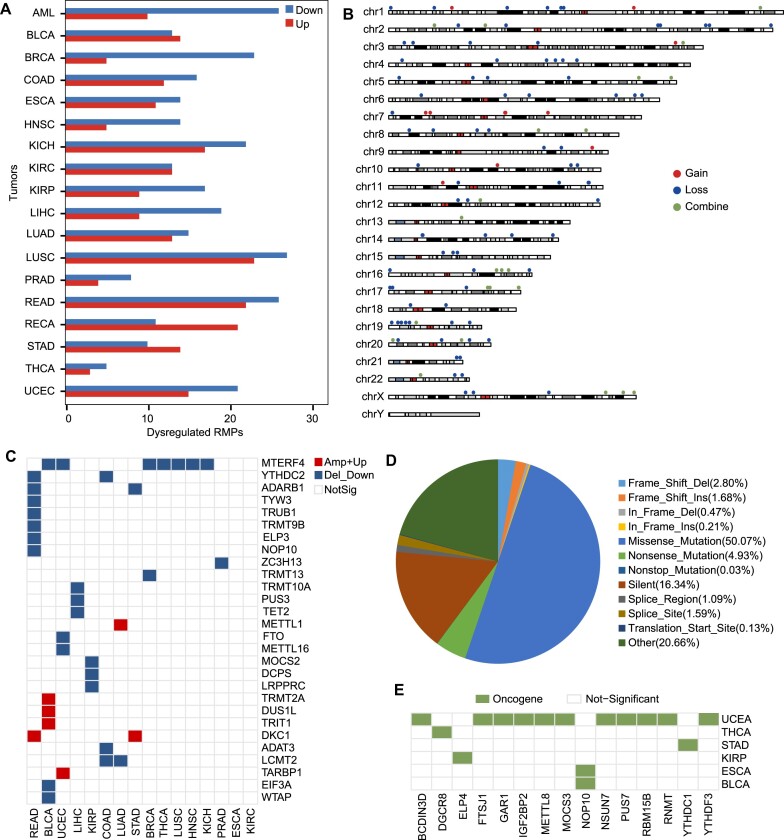
Exploration of dysregulated expression profiles and mutation maps of RMPs in tumors. (**A**) Barplot shows the up-regulated (red) and down-regulated RMPs in 18 types of tumors. (**B**) Chromosome plot displays the distributions of RMPs with recurrent SCNA in the genome. Red indicates RMPs gain extra copy number(s). Blue indicates RMPs lose copy number(s). Green indicates RMPs displays copy number gains or losses in different tumor types. (**C**) Heatmap shows recurrent SCNA-driver RMPs in 16 types of tumors in RMBase v3.0. (**D**) Distribution of diverse somatic mutations occurred on RMPs. (**E**) Somatic mutation-driver RMPs on diverse tumors in RMBase v3.0.

RNA-modifying proteins (RMPs), including RBPs that catalyze (Writer), remove (Eraser) and recognize (Reader) RNA modifications, whose dysregulation and mutation profoundly influence the occurrence and development of various cancers (62,63). To determine the potential function of RNA modifications in tumors, we systematically elucidated the abnormal expression profiles and mutation patterns of 146 RMPs in 18 types of tumors (Materials and methods). Differential expression profiles revealed dysregulated RMPs in all 16 types of tumors, with 85.6% (125/146) of RMPs showing up-regulation or down-regulation in most tumors (Figure 4A). Moreover, we identified that 76 RMPs are consistently down-regulated in the majority of tumors, while 49 tend to be up-regulated in most cases (Supplementary Figure S4). These findings suggest that RMP genes are often abnormally expressed and more prone to downregulation in tumor, although up-regulated RMPs also have important regulatory roles. For example, METTL1, an up-regulated RMP in COAD, LUAD, LUSC, READ and STAD, consistent with its roles of reducing the migration ability of cancer cells by maintaining its high levels in COAD, LUAD and LUSC (17).

Given that recurrent somatic copy number alterations (SCNAs) and somatic mutations are often the result of positive tumor selection (64,65), we constructed the mutation maps of 146 RMPs by deeply mined SCNA regions and somatic mutations in 16 types of tumors derived from TCGA. Here, we detected a total of 178 unique SCNA regions contained RMPs, consisting of 36 amplification regions and 142 deletion regions (Supplementary Table S6). We found that 84% (123/146) of RMPs are located within these SCNA regions (Figure 4B).

Notably, 12 RMPs were found in recurrent SCNA amplification regions, 86 RMPs in recurrent SCNA deletion regions, and 25 RMPs in various types of recurrent SCNA regions across different types of tumors. Remarkably, 38 RMPs, whose SCNA type is consistent with the dysregulation trend, were considered as potential SCNA-driver RMPs (Figure 4C). For instance, FTO with significantly down-regulated trend located in a deletion region, while up-regulated DKC1 located in an amplified SCNA region (Figure 4C). Moreover, we identified 14 818 non-redundant individual mutations in 146 RMPs. Somatic mutations were observed in all RMPs, with missense mutations representing the largest proportion (50.10%) (Figure 4D). We identified 16 somatic mutation-driver RMPs with significant somatic mutations in tumors (*q* ≤ 0.1) as candidate oncogene drivers in 6 different types of tumors (Figure 4E).

Although RMPs exhibit frequent aberrant expression and mutations in tumors, there are a relatively limited number of SCNA-driver RMPs and somatic mutation-driver RMPs (26% and 11%, respectively) that are determined by the aforementioned methods. This observation hints at the likelihood that the abnormalities and mutations of RMPs play a crucial regulatory role in the development of tumors as regulatory factors rather than driving factors.

### Web-based modules for exploring various RNA modifications

RMBase v3.0 provides 8 main modules to perform diverse sophisticated queries and display comprehensive results in a user-friendly manner. The ‘RNA Modifications’ module consists of 9 basic interfaces, namely m^1^A, m^5^C, m^6^A, m^7^G, 2′-O-Me, Ψ, RNA editing, ac^4^C and other types. It allows users to explore the landscape of RNA modifications in specific cell lines and provides detailed information for each RNA modification site, including genome coordinates, strand, gene biotypes, gene features, experimental datasets, PWM scores and biogenesis mechanisms related to enzymes or snoRNAs. Moreover, ‘modGene’ module provides a new page named ‘Clusters’ to investigate the cluster patterns of diverse RNA modifications based on their transcriptome maps in humans and mice. Combined with gene ontology, users can further explore potential biological functions of these clusters. An important aspect is the ‘Mechanisms’ module, which explores snoRNA-guided or enzyme-mediated RNA modifications. The ‘Browser’ page within this ‘Mechanisms’ module provides genome annotation and base-pairs for snoRNA targets. Furthermore, ‘Co-localization’ module deciphers the correlation of histone modifications and RNA modifications at the genome level. The ‘RMP’ module provides two query options, ‘RMP’ and ‘Cancer’, to explore the abnormal expression and mutations of RMPs in various tumor types. Apart from m^6^A, the ‘Motif’ module also performs the consensus sequence of other seven RNA modifications with motif logo, sequence, *P*-value, and other relevant information. The ‘Interactome’ module consist of ‘modRBP’, ‘modMirTar’ and ‘modVar’ that have been developed to investigates the interactome of RNA modifications with RBPs, miRNA targets, SNPs and SNVs (66). The ‘Tools’ module offers three powerful web-based tools: modAnnotation, modMetagene and modGeneTool that allow users to analyze their RNA modifications.

RMBase v3.0 also provided two user-friendly interfaces, ‘Download’ and ‘Help’, which allow users to download all data stored in the database. In addition, users can access the description pages of each module from the homepage to gain insights into the raw data information and summary results offered by the RMBase.

## Discussion and conclusions

In this study, RMBase v3.0 employed multiple standardized methods and pipelines to decode the most comprehensive landscape, potential mechanism and functions of 73 types of RNA modifications across 62 species (Figure 1, Table 1, Supplementary Table S7). In comparison to other databases and our previous release version (RMBase v2.0) (6,66), RMBase v3.0 showcases significant advancements and improvements (Supplementary Table S7), which are outlined as follows: (i) RMBase v3.0 expended RMBase v2.0 by integrating 1880 high-throughput epitranscriptomic sequencing data and a large amount of RNA modification sites for 62 species. It included 73 types of RNA modifications, including newly added three modifications collected from high-throughput sequencing data: m^7^G, ac^4^C and RNA editing. (ii) RMBase v3.0 systematically investigated the targeting relationships and regulatory mechanisms between snoRNAs with Ψ and 2′-*O*-Me (Figure 2), enhancing our comprehension of their biogenesis. (iii) RMBase v3.0 revealed the potential co-localization relationships of histone modifications with RNA modifications from a new perspective (Figure 3). (iv) RMBase v3.0, for the first time, explored dysregulated RMP expression and mutation maps in tumors, offering valuable insights into RNA modification's roles in tumor development. (v) RMBase v3.0 provided the RNA modification clusters in the human and mouse. (vi) RMBase v3.0 explored the relationships between RNA modifications with RBPs in six species, and with miRNA targets that were categorized into five classes including circRNAs, lncRNAs, mRNAs, pseudogenes and sncRNAs in human and mouse. (vii) RMBase v3.0 provided a new web-based tool named ‘modGeneTool’ to explore the modification maps on the gene or transcript of interest. (viii) Additionally, RMBase v3.0, for the first time, introduced a novel feature by offering RNA secondary structure features near RNA modification sites (Supplementary Figure S5). Enrichment analysis reveal that the potential RNA structure motifs associated with m^1^A and Ψ in human, yeast, bacteria, and fly, consistent with previous report (67,68). While the secondary structure features of m^7^G and m^5^C are more obvious in bacteria and fly. RMBase v3.0 improved the retrieval system and provided a variety of new web modules, graphic visualizations and tools to facilitate in-depth analyses and explorations of the massive RNA modifications.

However, there are some areas that still require improvement. This includes the exploration of RNA modification sequence features, distribution characteristics, and their associations with interacting factors in a cell- or tissue-specific manner, as well as the refinement of the analysis workflow to accommodate the *de novo* analysis of various RNA modification sequencing data including m^6^A, and more.

Overall, RMBase v3.0 strives to serve as a versatile platform for customized studies and is poised to become the standard repository for RNA epitranscriptome community.

## Future directions

As high-throughput epitranscriptome sequencing technology advances, an array of RNA modification sequencing technologies have been developed and applied. Our aim is to continually enhance the completeness of our database by incorporating these new data types in the future. RMBase will undergo further expansion to include additional RNA modification types, extended annotations, and a broader range of species. We will continue to integrate new RNA modification datasets, develop more efficient computational pipelines to discover their potential mechanisms and functions. Our ultimate goal is to maintain RMBase as a valuable and comprehensive resource in the field of RNA modification studies.

## Supplementary Material

gkad1070_Supplemental_File

## Data Availability

RMBase v3.0 is freely available at http://bioinformaticsscience.cn/rmbase. All of the data files can be downloaded and used in accordance with the GNU Public License and the licenses of the primary data sources.
