# Supplementary material for: RMBase v3.0: decode the landscape, mechanisms and functions of RNA modifications
Source: Nucleic Acids Res. 2023 Nov 13;52(D1):D273–84. doi: 10.1093/nar/gkad1070 (PMC10767931; doi:10.1093/nar/gkad1070)
Supplement: gkad1070_Supplemental_File [file gkad1070_supplemental_file.pdf]

Supplementary Material

Title:

RMBase v3.0: Decode the Landscape, Mechanisms and Functions of RNA Modifications

Authors:

Jiajia Xuan[1,2,*], Lifan Chen[1], Zhirong Chen[1], Junjie Pang[1], Junhong Huang[1], Jinran Lin[1,3], Lingling Zheng[1], Bin Li[1,*], Lianghu Qu[1,*], Jianhua Yang[1,*]

Figures

Figure S1. Global map of the RNA modifications in humans. (A) Heatmap of RNA modifications in different RNA species. The cell value represents the proportion of a particular RNA modification on an RNA to the total of that RNA modifications. Each row name represents one RNA modification type and its total number. Color bar shows the percentage. **(B)** Pie plot shows the abundance of clusters in five different RNA modifications. **(C)** Pie plot shows the distribution abundance of m^{6}A modification clusters on different RNA.

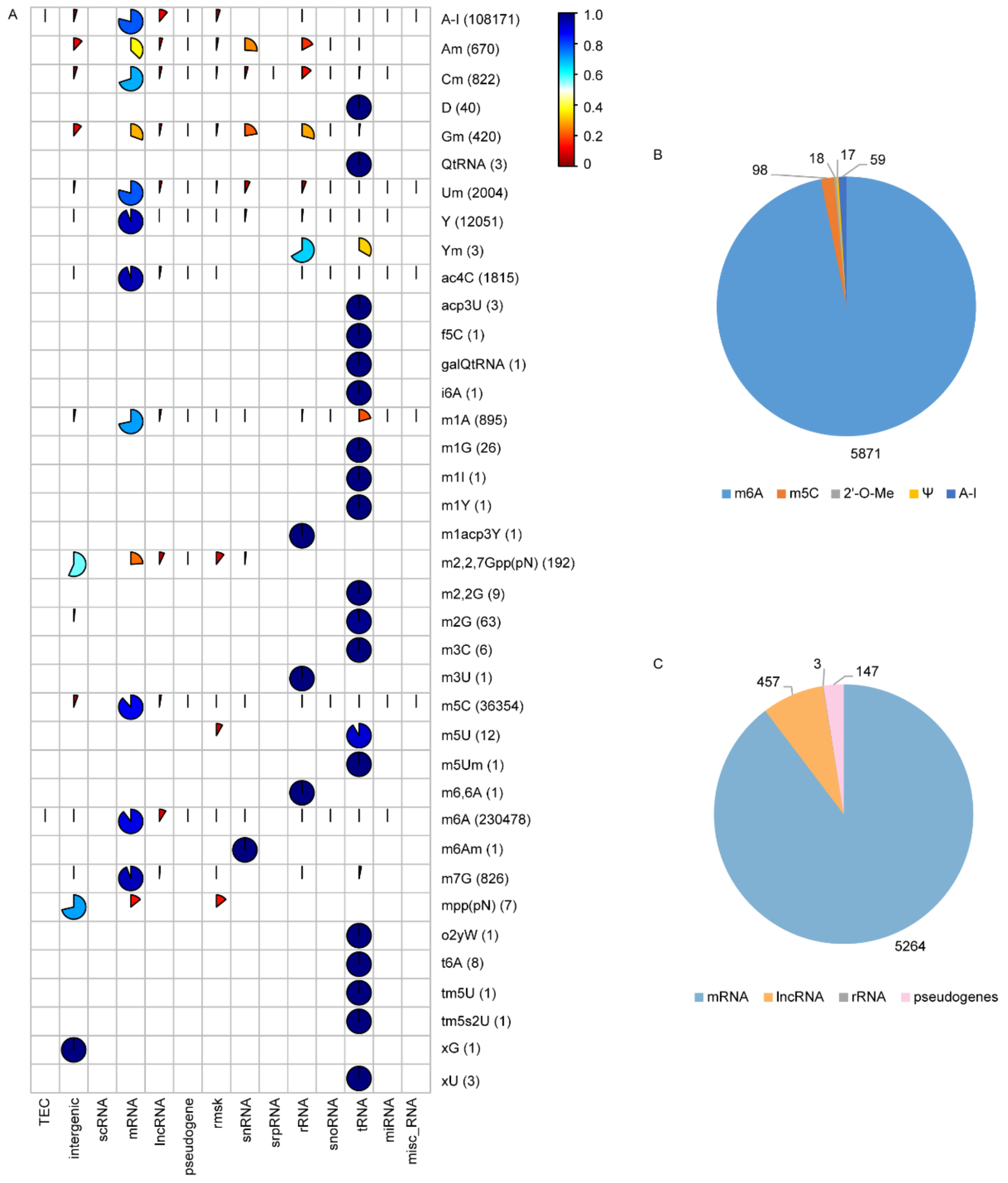

Figure S2. The metagene plots of different RNA modifications. These plots for m^6A modification (A), A-I modification (B), Ψ modification (C) and m^5C modification (D) in different species are generated using the "modMetagene" tool provided by RMBase v3.0.

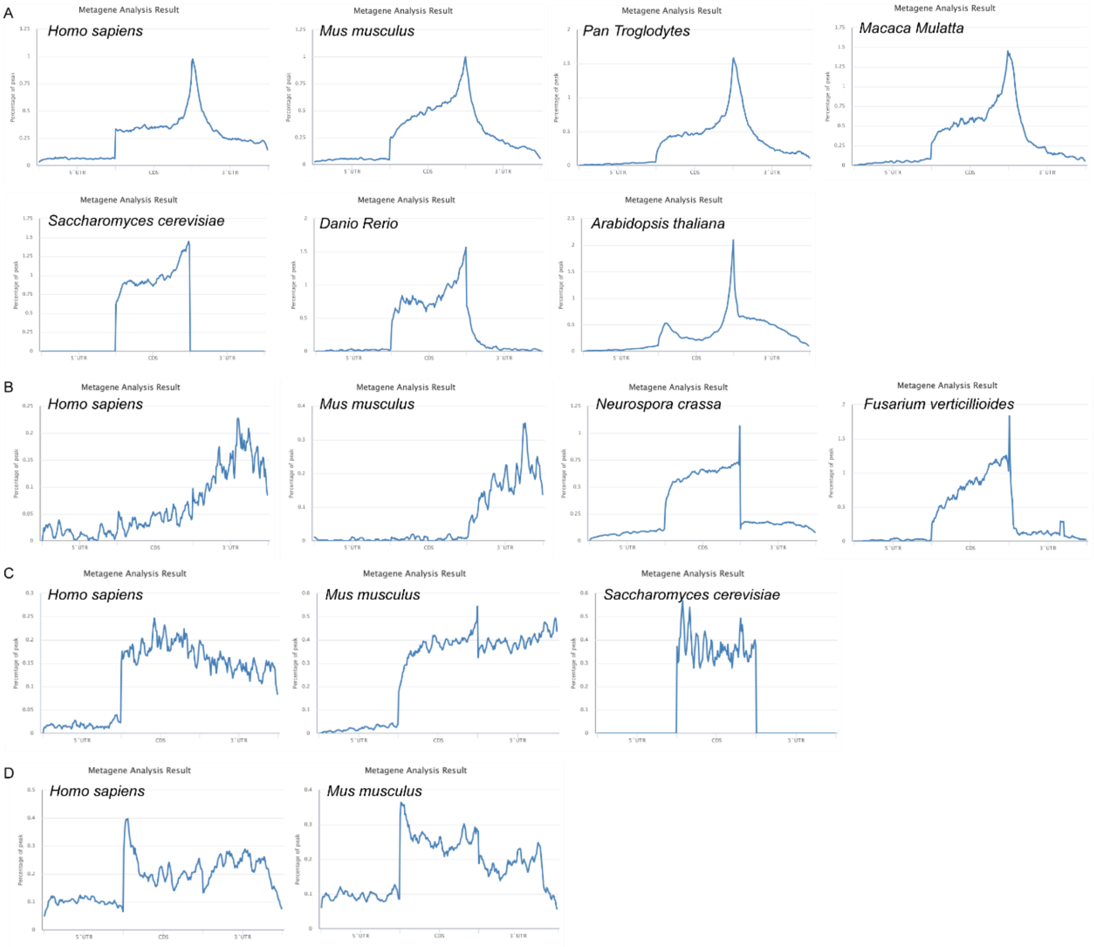

Figure S3. Identification of 2'-O-Me modification sites guided by snoRNAs using sno2Nm. (A) Flowchart of sno2Nm. **(B)** Pie plot shows that the distribution of known and novel snoRNA-guided 2'-O-Me sites on different gene biotypes.

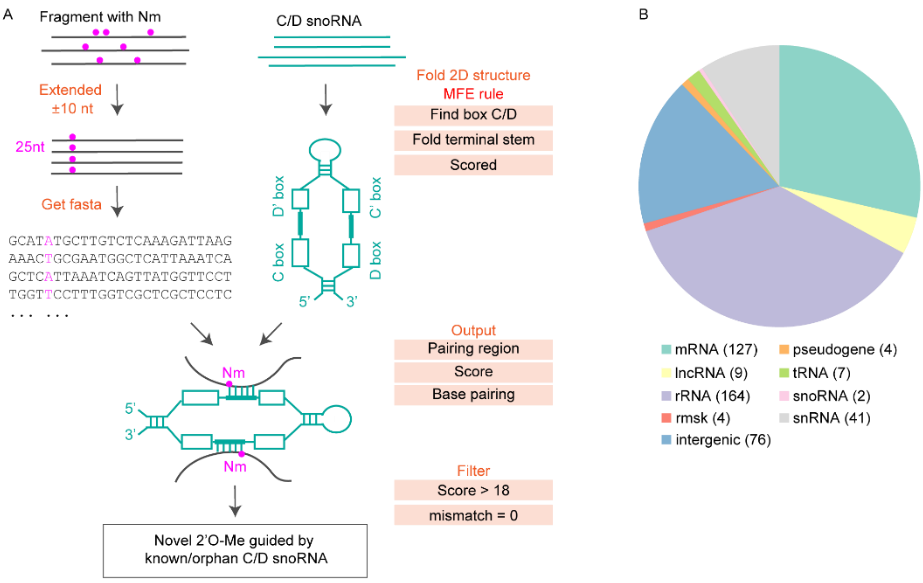

Figure S4. Heatmap for differential expression of RMPs in 16 types of tumors. "red" refers to up-regulated RMPs in tumors, while "blue" refers to down-regulated RMPs in tumors.

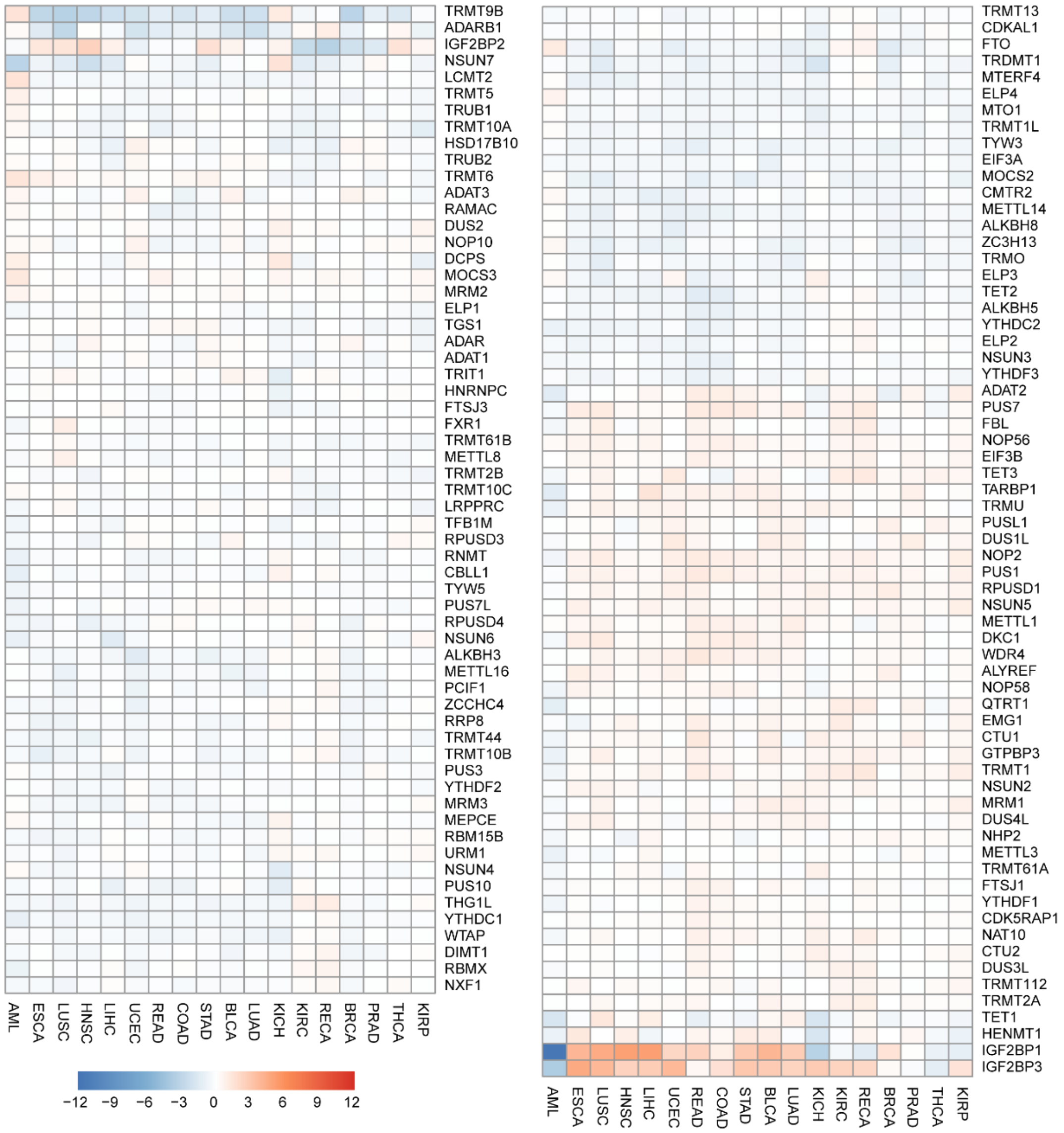

Figure S5 The potential RNA structure motifs associated with specific RNA modifications. The seqlogo plots show the RNA secondary structure features near the m^1A modification site (A), Ψ modification site (B), m^7G modification site (C) and m^5C modification site (D) across different species. Where, "C" stands for "(", "G" stands for ")", and "T" stands for "." in the MFE secondary structure (dot-bracket). The modification site is at the sixteenth position.

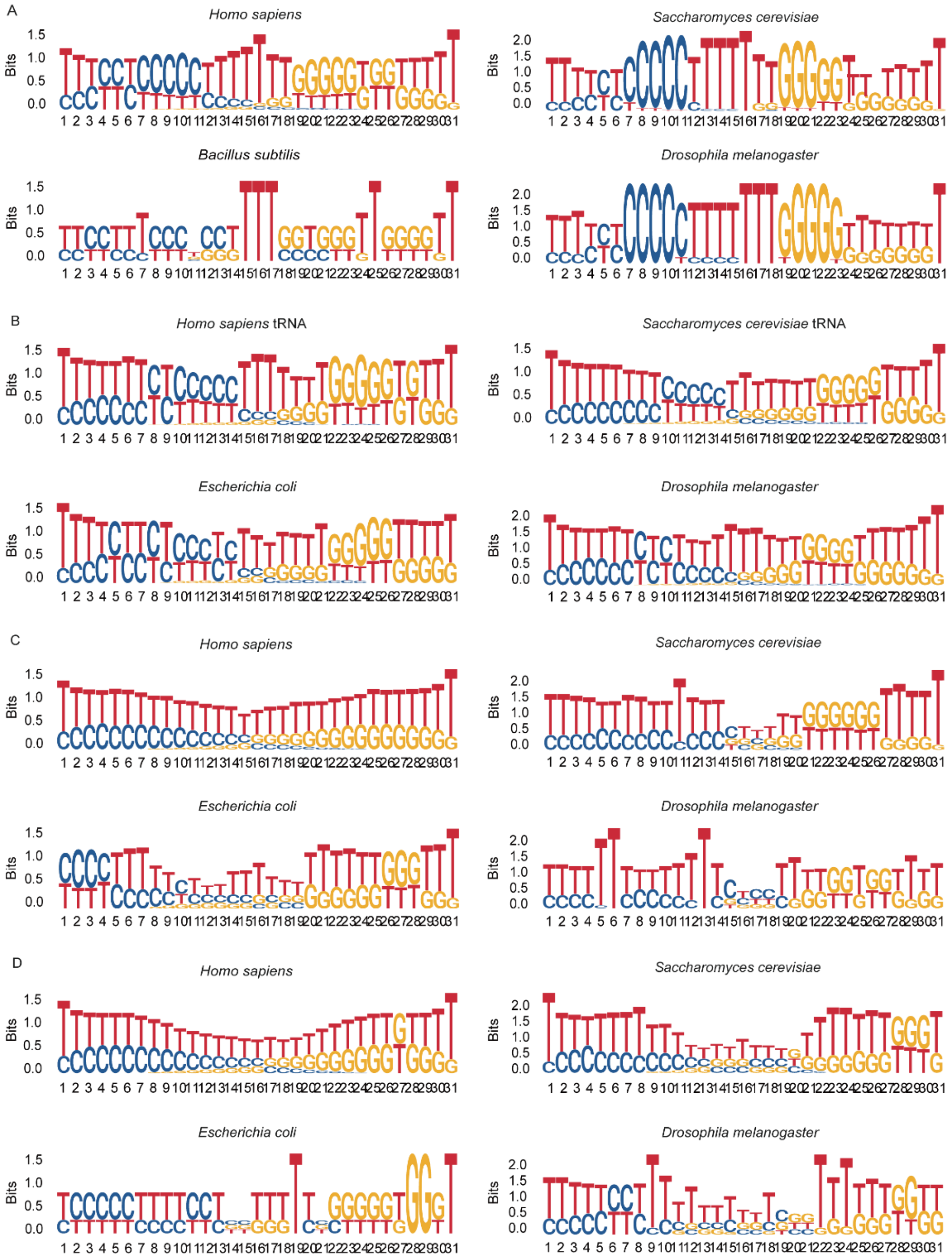

Tables

Table S1. Datasets source of RNA modifications and their intersecting factors.

Data	Datasets Source	Datasets List
N^1-methyladenosine (m^1A)	GEO, PubMed	m1A-seq, m1A-MAP
N^5-methylcytosine (m^5C)	GEO, PubMed	m5C-RIP, Bisulfite-seq
N^6-methyladenosine (m^6A)	GEO, PubMed	miCLIP, m6A-CLIP/IP, MAZTER-seq, DART-seq, m6A-REF-seq, m6A-seq, MeRIP-seq
N^7-Methylguanosine (m^7G)	GEO, PubMed	m7G-seq, BoRed-seq, m7G-RIP-seq
Pseudouridine (Ψ)	GEO, PubMed, snOPY, Yeast-snoRNADataBase, snoRNABase	Ψ-seq, CeU-seq, Pseudo-seq, PSI-seq, BID-seq, PRAISE
2′-O-methylation (2'-O-Me)	GEO, PubMed, snOPY, Yeast-snoRNADataBase, snoRNABase	Nm-seq, RiboMeth-seq, RibOxi-seq, 2OMe-seq
N^4-acetylcytidine (ac^4C)	GEO	ac4C-seq, acRIP-seq
RNA-editing (A-I)	FairBase, REDIportal, RADAR and DARNED	/
Other modifications	MODOMICS	/
RNA binding proteins (RBP)	starBase (1), ENCODE and POSTAR2 (2)	eCLIP, HITS-CLIP, iCLIP, PARCLIP
miRNA targets	starBase	/
single-nucleotide variations (SNV)	COSMIC (3) and published literatures (4,5)	/
single-nucleotide polymorphisms (SNP)	Clinvar (6), GWAS (7) and GTEx (8)	/

Table S2. Source of genomes and gene annotations of 62 species.

Group	Species	Assembly	Genome and Annotations
Mammal	*Homo sapiens*	hg38	gencode.v30, UCSC, miRBase v22
	Mus musculus	mm10	gencode.vM22, UCSC, miRBase v22
	Macaca mulatta	rheMac8	Ensembl v97, UCSC, miRBase v22
	Pan troglodytes	panTro5	Ensembl v97, UCSC, miRBase v22
	Rattus norvegicus	rn6	Ensembl v97, UCSC, miRBase v22
	Sus scrofa	susScr11	Ensembl v97, UCSC, miRBase v22
	Mesocricetus auratus	MesAur1.0	Ensembl v97
	Oryctolagus cuniculus	OryCun2.0	Ensembl v97, UCSC, miRBase v22
	Ovis aries	Oar_v3	Ensembl v97, UCSC, miRBase v22
	Bos taurus	bosTau9	Ensembl v97, UCSC, miRBase v22
Plant	*Arabidopsis thaliana*	TAIR10	Ensembl v44, GtRNAdb v18, miRBase v22
	Brassica napus	AST_PRJEB5043_v1	Ensembl v44
	Chlamydomonas reinhardtii	CHLRE3	NCBI
	Cucumis sativus	ASM407v2	Ensembl v44
	Glycine max	glyMax2.1	Ensembl v44, GtRNAdb v18, miRBase v22
	Nicotiana tabacum	Ntab-TN90	NCBI
	Phaseolus vulgaris	PhaVulg1	Ensembl v44, miRBase v22
	Solanum tuberosum	SolTub3	Ensembl v44, miRBase v22
	Spinacia oleracea	ASM200726v1	NCBI
	Zea mays	RefGenv4	Ensembl v44
Metazoa	*Bombyx mori*	ASM15162v1	Ensembl v44
	Caenorhabditis elegans	WBcel235	Ensembl v97, UCSC, miRBase v22
	Xenopus laevis	Xenopus_laevis_v2	NCBI
Vertebrate	*Danio rerio*	danRer11	Ensembl v97, UCSC, miRBase v22
	Gallus gallus	GRCg6a	Ensembl v97, UCSC, miRBase v22
Fungi	*Candida albicans*	Cand_albi_12C	Ensembl v44
	Fusarium graminearum	ASM24013v3	Ensembl v45, GtRNAdb v18
	Fusarium verticillioides	ASM14955v1	Ensembl v45, GtRNAdb v18
	Neurospora crassa	NC12	Ensembl v44
	Pichia jadinii	Cybja1	NCBI
	Saccharomyces cerevisiae	sacCer3	Ensembl v97, GtRNAdb v18
	Schizosaccharomyces pombe	ASM294v2	Ensembl v44, GtRNAdb v18
Insect	*Drosophila melanogaster*	BDGP6	Ensembl v97, UCSC, miRBase v22
Protists	*Tetrahymena thermophila*	JCVI-TTA1-2.2	Ensembl v44
Bacteria	*Bacillus subtilis*	ASM69118v1	Ensembl v44

	Clostridium acetobutylicum	ASM78585v1	Ensembl v44
	Escherichia coli	ASM584v2	Ensembl v44, GtRNAdb v18
	Geobacillus stearothermophilus	ASM74998v1	Ensembl v44
	Halobacterium salinarum	ASM680v1	Ensembl v44, GtRNAdb v18
	Halococcus morrhuae	ASM33669v1	Ensembl v44
	Haloferax volcanii	ASM2568v1	Ensembl v44, GtRNAdb v18
	Lactococcus lactis	ASM76111v1	Ensembl v44, GtRNAdb v18
	Mycobacterium smegmatis	ASM76770v1	Ensembl v44, GtRNAdb v18
	Mycoplasma capricolum	ASM83033v1	Ensembl v44, GtRNAdb v18
	Mycoplasma mycoides	ASM25307v1	Ensembl v44, GtRNAdb v18
	Pseudomonas aeruginosa	ASM676v1	Ensembl v44, GtRNAdb v18
	Pyrococcus abyssi	ASM19593v2	Ensembl v45
	Rhodospirillum rubrum	ASM1308v1	Ensembl v44
	Salmonella typhimurium	ASM553737v1	NCBI
	Spiroplasma citri	ASM188685v1	NCBI
	Streptomyces coelicolor A3 (2)	ASM20383v1	Ensembl v44
	Streptomyces griseus	ASM93222v1	Ensembl v44
	Sulfolobus acidocaldarius	ASM1228v1	Ensembl v44, GtRNAdb v18
	Sulfolobus solfataricus	ASM96839v1	Ensembl v44
	Synechococcus elongatus PCC 6301	ASM1006v1	NCBI, GtRNAdb v18
	Synechococcus sp.PCC 7002	ASM1948v1	NCBI, GtRNAdb v18
	Synechocystis sp	ASM34078v1	NCBI
	Thermoplasma acidophilum	ASM19591v1	Ensembl v44, GtRNAdb v18
	Thermotoga maritima	ASM97857v1	Ensembl v44, GtRNAdb v18
	Thermus thermophilus	ASM812v1	Ensembl v44, GtRNAdb v18
Virus	*Enterobacteria phage T4*	ViralProj14044	NCBI
	Enterobacteria phage T5	ASM292140v1	NCBI

Table S3. Overview of the top five clusters with the most abundant RNA modification sites of m^{6}A, m^{5}C, 2'-O-Me, Ψ, and A-I. "Number" is the number of RNA modification sites in the cluster. "Density" refers to the average distance between RNA modification sites within a cluster.

Gene Name	Gene Type	Cluster	Number	Density	Modification
MKI67	mRNA	chr10:128105818-128109436:-	113	32.0177	m6A
MKI67	mRNA	chr10:128103187-128105817:-	83	31.6867	m6A
PLIN4	mRNA	chr19:4510671-4513028:-	75	31.4267	m6A
AL121827.2	lncRNA	chr20:63256960-63258120:+	74	15.6757	m6A
CREB3L3	mRNA	chr19:4171681-4172987:+	71	18.3944	m6A
CENPB	mRNA	chr20:3786441-3786639:-	29	6.82759	m5C
ANKRD9	mRNA	chr14:102509599-102509799:-	25	8	m5C
ZSWIM6	mRNA	chr5:61332733-61332870:+	24	5.70833	m5C
BTBD2	mRNA	chr19:2015393-2015714:-	21	15.2857	m5C
TBL1X	mRNA	chrX:9465148-9465351:+	20	10.15	m5C
FP236383.1	lncRNA	chr21:8400486-8401014:+	23	22.9565	2'-O-Me
FP236383.1	lncRNA	chr21:8444718-8445246:+	23	22.9565	2'-O-Me
FP671120.2	lncRNA	chr21:8217522-8217976:+	21	21.619	2'-O-Me
FP236383.1	lncRNA	chr21:8401055-8401597:+	19	28.5263	2'-O-Me
FP236383.1	lncRNA	chr21:8445287-8445829:+	19	28.5263	2'-O-Me
FP671120.2	lncRNA	chr21:8218114-8218611:+	20	24.85	Ψ
FP236383.1	lncRNA	chr21:8401152-8401649:+	20	24.85	Ψ
FP236383.1	lncRNA	chr21:8445384-8445881:+	20	24.85	Ψ
FP671120.2	lncRNA	chr21:8210380-8211125:+	17	43.8235	Ψ
FP236383.1	lncRNA	chr21:8393415-8394160:+	17	43.8235	Ψ
BMS1P10	pseudogene	chr9:63302059-63302732:-	59	11.4068	A-I
OSMR-AS1	lncRNA	chr5:38820960-38821768:-	41	19.7073	A-I
ISPD	mRNA	chr7:16089130-16089520:-	38	10.2632	A-I
TNFRSF14-AS1	lncRNA	chr1:2556076-2556686:-	32	19.0625	A-I
OSMR-AS1	lncRNA	chr5:38822949-38823463:-	31	16.5806	A-I

Table S4. The Ψ targets of H/ACA snoRNAs (top 15 for guide H/ACA snoRNA and orphan H/ACA snoRNA respectively). "[a]" refers to known guide H/ACA snoRNA, "[b]" refers to orphan H/ACA snoRNA.

snoRNA	Modification Site	Score	Target Type	Gene Type	Gene	Target Sequence
SNORA16A [a]	28S:4412	27.332	known	rRNA	LSU-rRNA_Hsa	CGCUUUUUGAYCCUUCGAUGU
SNORA31 [a]	28S:3713	26.462	known	rRNA	LSU-rRNA_Hsa	AGAAAUUCAAYGAAGCGCGGG
SNORA17A [a]	28S:4659	25.871	known	rRNA	LSU-rRNA_Hsa	GGAUUAUGACYGAACGCCUCU
SNORA27 [a]	28S:3694	25.392	known	rRNA	LSU-rRNA_Hsa	GCUCUGAAUGYCAAAGUGAAG
SCARNA3 [a]	U6:40	24.733	known	snRNA	U6	AUUGGAACGAYACAGAGAAGA
SNORA8 [a]	18S:1081	24.046	known	rRNA	SSU-rRNA_Hsa	GUUCCGACCAYAAACGAUGCC
SNORA17A [a]	psi_2154	23.326	unknow	rRNA	LSU-rRNA_Hsa	GGAUUAUGACYGAACGCCUUU
SNORA68B [a]	psi_2197	23.157	unknow	lncRNA	MAGI2-AS3	UUCUGUUUAAYAUCUUCAAGU
SNORA68 [a]	psi_2197	23.157	unknow	lncRNA	MAGI2-AS3	UUCUGUUUAAYAUCUUCAAGU
SNORA2A [a]	psi_2258	22.992	unknow	mRNA	C9orf85	UAGAUUUUGAYAUUGAUUUAG
SNORA10B [a]	psi_151	22.718	unknow	mRNA	C11orf1	ACCUGAGGAAYAUCGUGCAGC
SNORA10 [a]	psi_151	22.718	unknow	mRNA	C11orf1	ACCUGAGGAAYAUCGUGCAGC
SNORA30 [a]	28S:4643	22.557	known	rRNA	LSU-rRNA_Hsa	GAAGCUACCAYCUGUGGGAUU
SNORA3B [a]	28S:3899	22.557	known	rRNA	LSU-rRNA_Hsa	CCUGUUGAGCYUGACUCUAGU
SNORA30B [a]	28S:4643	22.557	known	rRNA	LSU-rRNA_Hsa	GAAGCUACCAYCUGUGGGAUU
SNORA2_3 [b]	psi_2258	22.992	unknow	mRNA	C9orf85	UAGAUUUUGAYAUUGAUUUAG
SNORA38B [b]	28S:4269	22.869	known	rRNA	LSU-rRNA_Hsa	UUGAUCUUGAYUUUCAGUACG
SNORA101B [b]	psi_1561	21.335	unknow	snRNA	U2	CUUCUCGGCCYUUUGGCUAAG
SNORA58B [b]	psi_2313	21.008	unknow	mRNA	LAP3	UGAAGUUCCCYAUCUACGGAA
SNORA15B-1 [b]	psi_2031	20.947	unknow	mRNA	ZNF75D	AGCUGUCUGUYUUAUUCAGCA
SNORA15B-2 [b]	psi_2031	20.947	unknow	mRNA	ZNF75D	AGCUGUCUGUYUUAUUCAGCA
SNODB2082 [b]	psi_319	20.936	unknow	mRNA	PHGDH	CAGAGUGGAGYCUCCUCACAG
SNORA7 [b]	28S:1779	20.368	known	rRNA	LSU-rRNA_Hsa	UCUCAAACUUYAAAUGGGUAA

SNORA58B [b]	28S:3823	20.342	known	rRNA	LSU-rRNA_Hsa	CCACUGUCCCYACCUACUAUC
SNORA5 [b]	18S:1625	19.842	known	rRNA	SSU-rRNA_Hsa	UUGCAAUUAUYCCCCAUGAAC
SNORA5 [b]	psi_648	19.842	unknow	rRNA	SSU-rRNA_Hsa	UUGCAAUUAUYCCCCAUGAAG
SNORA5 [b]	psi_1951	19.842	unknow	rRNA	SSU-rRNA_Hsa	UUGCAAUUAUYCCCCAUGACC
SNORA38 [b]	psi_147	19.813	unknow	mRNA	MRPS21	UCCAUCUCUUYUCUUUGUACA
SNORA38 [b]	psi_1486	19.813	unknow	mRNA	PEX1	CUGCAUUCUUYUCUUUGAUGA
SNORA38 [b]	psi_1609	19.813	unknow	mRNA	PPP4R1	GACAGCUCUUYACUUUGUACU

Table S5. The 2'-O-Me targets of C/D snoRNAs (top 20).

snoRNA	Gene Type	Gene	Target Sequence	Score
SNORD57	intergenic	intergenic	GCTCATTAAATCAGTTTTTTTGCCT	45.236
SNORD20	rRNA	SSU-rRNA_Hsa	AACTTGACTATCTAGAGGAA	36.508
SNORD14E	rRNA	SSU-rRNA_Hsa	ACATCCAAGGAAGGTAG	31.878
SNORD59B	intergenic	intergenic	AAGAACGAAAGTCAGAA	31.145
SNORD91B	rRNA	LSU-rRNA_Hsa	ACAGGTTCAGACATTT	29.355
SNORD91B	rRNA	LSU-rRNA_Hsa	ACAGGTTCAGACATTT	29.355
SNORD57	rRNA	SSU-rRNA_Hsa	GCTCATTAAATCAGTT	28.968
SNORD37	intergenic	intergenic	GTCAAAGTGAAGAAAT	28.912
SNORD14A	intergenic	intergenic	ACATCCAAGGAAGGACT	28.456
SNORD14A	mRNA	KCTD10	TCATCCAAGGAAGAAC	28.274
SNORD14B	rRNA	SSU-rRNA_Hsa	ACATCCAAGGAAGGT	28.131
SNORD14C	rRNA	SSU-rRNA_Hsa	ACATCCAAGGAAGGT	28.131
SNORD91B	rRNA	LSU-rRNA_Hsa	GCAGGTTCAGACATTT	27.652
SNORD91B	rRNA	LSU-rRNA_Hsa	CCAGGTTCAGACATTT	27.652
SNORD67	snRNA	U6	ATGGCCCCTGTGCA	27.258
SNORD37	mRNA	CNTNAP5	GTCAAAGTGAAGAAA	27.255
SNORD37	pseudogene	AC109583.1	GTCAAAGTGAAGAAA	27.255
SNORD45A	mRNA	CAMK2D	TGTAATTCTAGAGCTA	27.214
SNORD45C	mRNA	CAMK2D	TGTAATTCTAGAGCTA	27.214
SNORD105	rRNA	SSU-rRNA_Hsa	AGCGTTTACTTTGAA	27.168

Table S6. Overview of SCNA regions, consist of 36 amplification regions (Any-AP*) and 142 deletion regions (Any-DP*), that are identified from 146 RMPs in 16 types of tumors.

SCNA ID	Genome Coordinate	SCNA ID	Genome Coordinate	SCNA ID	Genome Coordinate
Any-DP22	chr10:18039971-38011171	Any-DP30	chr15:23687036-47187107	Any-DP4	chr2:234076904-242193529
Any-AP10	chr10:67553187-83045635	Any-DP34	chr15:33847092-43414476	Any-DP1	chr2:238550823-242193529
Any-DP16	chr10:84516936-133797422	Any-DP31	chr15:35232510-57223137	Any-DP4	chr2:238552211-242193529
Any-DP25	chr10:84517918-133797422	Any-DP33	chr15:37092607-43751053	Any-DP4	chr2:240605436-242193529
Any-DP23	chr10:99227306-133797422	Any-DP35	chr15:37099383-65387736	Any-DP31	chr22:43808440-50818468
Any-DP25	chr11:103083226-133786067	Any-DP3	chr1:54882224-146716040	Any-DP45	chr22:45147014-50818468
Any-DP28	chr11:104248923-135086622	Any-DP3	chr1:60968430-146716040	Any-DP43	chr22:45598443-50818468
Any-DP17	chr11:106051237-135086622	Any-DP11	chr16:1073224-90338345	Any-DP40	chr22:45842244-50818468
Any-DP29	chr11:107017966-122090888	Any-DP34	chr16:1-1772130	Any-AP4	chr2:27050024-31902797
Any-DP2	chr1:1-22273002	Any-DP37	chr16:1-860688	Any-DP4	chr2:29462731-119170220
Any-DP2	chr1:1-27537744	Any-DP40	chr16:31758063-70219917	Any-DP5	chr2:32038092-47339841
Any-DP2	chr1:1-27540195	Any-AP27	chr16:67051986-77286707	Any-DP6	chr2:40508595-45657277
Any-DP3	chr1:1-31503462	Any-DP17	chr16:74413389-90338345	Any-AP3	chr2:60217741-61040694
Any-DP2	chr1:1-32184201	Any-DP18	chr16:75653775-90338345	Any-AP6	chr3:162740552-198295559
Any-AP17	chr11:33521255-35407436	Any-DP34	chr16:85305209-90338345	Any-AP1	chr3:175206052-198295559
Any-AP21	chr11:34140379-35099383	Any-DP35	chr17:1-1921251	Any-DP6	chr3:183399451-198295559
Any-DP1	chr1:1-34868247	Any-DP44	chr17:1-4276191	Any-DP5	chr3:1-99843146
Any-DP18	chr11:37945516-49038091	Any-DP12	chr17:1-9804657	Any-DP5	chr3:42221061-64095595
Any-AP1	chr1:153772474-155317730	Any-DP28	chr17:40859914-83257441	Any-DP5	chr3:45787059-62264083
Any-DP3	chr1:1-5865449	Any-AP10	chr17:53482168-83257441	Any-DP8	chr4:1-12228847
Any-DP2	chr1:16449987-32474850	Any-AP30	chr17:58972171-82613252	Any-DP8	chr4:113024717-133099621
Any-DP1	chr1:1-8210429	Any-AP22	chr17:70437748-83257441	Any-DP8	chr4:68339322-99412774
Any-DP13	chr11:85844359-135086622	Any-DP36	chr17:80961398-83257441	Any-DP10	chr4:94736854-112153735

Any-DP14	chr11:96375540-135086622	Any-DP3	chr1:78854116-146716040	Any-DP9	chr5:103557581-114366226
Any-DP25	chr12:118416942-133275309	Any-DP19	chr18:1-80373285	Any-DP14	chr5:109877935-115172890
Any-DP29	chr12:121417330-133275309	Any-DP36	chr18:36065361-74410170	Any-DP4	chr5:1208683-136317879
Any-DP20	chr12:123244069-133275309	Any-DP2	chr1:84139647-115055946	Any-AP6	chr5:121373929-181538259
Any-DP19	chr12:23188659-133275309	Any-DP3	chr1:89934392-102877252	Any-AP5	chr5:151548708-181538259
Any-DP1	chr1:224734013-237051607	Any-DP36	chr19:1100076-1943799	Any-DP11	chr5:155014906-181538259
Any-AP5	chr1:234376848-235100677	Any-DP29	chr19:1-11096366	Any-DP9	chr5:31177078-181538259
Any-AP17	chr12:57692920-57857873	Any-DP41	chr19:1-1912474	Any-DP10	chr5:75487100-143813640
Any-AP15	chr12:57739474-57976149	Any-DP20	chr19:1271276-4232571	Any-DP14	chr5:78789805-135040865
Any-DP30	chr12:69954235-133275309	Any-DP41	chr19:1293079-2233892	Any-DP18	chr6:1-24429461
Any-DP29	chr12:72026299-133275309	Any-DP41	chr19:1-3366418	Any-DP10	chr6:128023474-170805979
Any-DP25	chr12:75427945-133275309	Any-DP13	chr19:1-57774763	Any-DP13	chr6:1-30499917
Any-DP27	chr12:98544935-133275309	Any-DP21	chr19:1-58617616	Any-DP14	chr6:149072252-170805979
Any-AP15	chr13:1-47260471	Any-AP43	chr19:16565517-18004678	Any-DP12	chr6:57309540-117487381
Any-DP10	chr13:22885862-86954491	Any-DP33	chr19:1-7401250	Any-DP17	chr6:57347010-132302870
Any-DP21	chr13:44548519-48305524	Any-DP42	chr19:19351525-58617616	Any-DP13	chr6:73828037-121080765
Any-AP2	chr1:38858493-43645853	Any-DP39	chr19:45484771-58617616	Any-DP12	chr6:79311053-98836429
Any-AP2	chr1:39574036-40078732	Any-DP1	chr1:98271211-119712502	Any-DP14	chr7:1-3302828
Any-AP1	chr1:39789450-40133402	Any-DP34	chr20:1-64444167	Any-AP7	chr7:21559416-37277484
Any-DP21	chr14:102722147-107043718	Any-AP46	chr20:2586165-4402978	Any-AP8	chr7:70337996-103926122
Any-DP29	chr14:103186698-107043718	Any-AP21	chr20:43613202-50341857	Any-DP13	chr8:11771885-51321318
Any-DP19	chr14:1-107043718	Any-DP37	chr21:18220890-46709983	Any-AP13	chr8:122611767-128894558
Any-DP33	chr14:1-28729025	Any-DP7	chr2:121285113-181676580	Any-DP16	chr8:15237757-29922546
Any-DP20	chr14:1-34433896	Any-DP2	chr2:1-242193529	Any-DP15	chr8:21895475-31643737
Any-DP15	chr14:1-34716226	Any-DP44	chr21:37267476-46709983	Any-DP17	chr8:22257058-28321147
Any-DP30	chr14:1-46069185	Any-DP41	chr21:39347707-43612563	Any-DP6	chr8:55763213-133244567

Any-DP30	chr14:1-49579565	Any-DP5	chr2:208822783-242193529	Any-AP11	chr8:64714595-113387382
Any-DP1	chr1:4418360-31368947	Any-DP44	chr22:1-21741481	Any-AP12	chr9:124783428-129198374
Any-DP21	chr14:57863913-107043718	Any-DP4	chr2:212536940-242193529	Any-DP21	chr9:38617174-111900370
Any-DP29	chr14:58274934-107043718	Any-DP1	chr2:213742524-242193529	Any-DP15	chrX:1-156040895
Any-DP29	chr14:81617875-107043718	Any-DP14	chr22:1-50818468	Any-AP24	chrX:133367616-156040895
Any-DP34	chr14:81618928-107043718	Any-DP4	chr2:217939472-242193529	Any-AP30	chrX:133560672-156040895
Any-DP33	chr14:92740008-107043718	Any-AP36	chr22:18124604-26431626	Any-AP28	chrX:152108541-156040895
Any-DP32	chr15:1-40693915	Any-AP30	chr22:18131163-21785126	Any-AP28	chrX:152576712-155653639
Any-DP16	chr15:1-56262145	Any-DP2	chr2:224394640-242193529	Any-AP50	chrX:153530695-155653639
Any-DP23	chr15:1-61854397	Any-DP3	chr2:227147251-242193529		
Any-DP35	chr15:1-82096090	Any-DP5	chr2:228174428-242193529		

Table S7. Improvements of RMBase v3.0 against previous versions.

This table describes the major improvements of data features and web-based functionalities in updated RMBase v3.0 by comparing to previous version. ([a] is Curated Data, [b] is Main Results, [c] is Web-based Functionalities)

Items	RMBase v3.0	previous version
Species [a]	62 species (9 clades)	13 species (6 clades)
Types of RNA Modifications [a]	73	47
Datasets of RNA Modifications [a]	1,886 datasets (834 WT datasets), 6 databases	604 datasets, 3 databases
Datasets of m^6A [a]	689 datasets, modomics	507 datasets, modomics
Datasets of m^1A [a]	72 datasets, modomics	72 datasets, modomics
Datasets of m^5C [a]	38 datasets, modomics	4 datasets, modomics
Datasets of pseudo [a]	19 datasets, modomics, snoRNABase, snOPY	16 datasets, modomics, snoRNABase, snOPY
Datasets of 2'-O-Me [a]	6 datasets, modomics, snoRNABase, snOPY	5 datasets, modomics
Datasets of m^7G [a]	6 datasets, modomics	modomics
Datasets of ac^4C [a]	4 datasets, modomics	modomics
Datasets of RNA-editing [a]	modomics, REDIportal, DARNED, RADAR, FairBase	modomics
RBP Datasets [a]	1,439 (6 species)	(2 species)
RBP Biding Sites (Raw Data) [a]	108,320,121	67,598,301
SNV Sites [a]	19,437,720	19,087,089
SNP Sites [a]	46,735,114	2,052,046
Types of miRNA Targets [a]	5	1
miRNA Targets (circRNA) [a]	1,229,615	0
miRNA Targets (lincRNA) [a]	83,759	0
miRNA Targets (mRNA) [a]	3,170,237	426,683
miRNA Targets (pseudogene) [a]	105,326	0
miRNA Targets (sncRNA) [a]	23,578	0
Histone Modifications [a]	14	0
RNA Modifications Sites [b]	2,545,031 (1,103,583 high accuracy sites)	1,397,244
RNA Modification Clusters [b]	6,068	0
Regularory Pairs of mod-mirTar (circRNA) [b]	258,111	0
Regularory Pairs of mod-mirTar (lincRNA) [b]	10,657	0
Regularory Pairs of mod-mirTar (mRNA) [b]	314,520	45,423

Regularory Pairs of mod-mirTar (pseudogene) [b]	7,746	0
Regularory Pairs of mod-mirTar (sncRNA) [b]	1,684	0
Regularory Pairs of mod-RBP binding sites [b]	7,178,019	3,261,544
Regularory Pairs of mod-SNVs [b]	971,808	192,283
Regularory Pairs of mod-SNPs [b]	3,904	1,862
Co-localization of RNA modifications and histone modification [b]	13 cells, 6 RNA modifications * 32 histone modifications	0
Expression profiles of RMPs in cancers [b]	Yes	No
somatic mutation-driver RMPs [b]	16	0
SCNA-driver RMPs [b]	38	0
Mechanism (snoRNA-guided Ψ and 2'-O-Me) [b]	Yes	No
Motif [b]	11 RNA modifications	m^6A and m^1A
RNA secondary structures [b]	Yes	No
Web-based Tools [c]	3	2
Cell/Tissues Information [c]	Yes	No
Evolutionary Conservation [c]	Yes	No

References

1. Li, J.H., Liu, S., Zhou, H., Qu, L.H. and Yang, J.H. (2014) starBase v2.0: decoding miRNA-ceRNA, miRNA-ncRNA and protein-RNA interaction networks from large-scale CLIP-Seq data. *Nucleic Acids Res*, **42**, D92-97.
2. Zhu, Y., Xu, G., Yang, Y.T., Xu, Z., Chen, X., Shi, B., Xie, D., Lu, Z.J. and Wang, P. (2019) POSTAR2: deciphering the post-transcriptional regulatory logics. *Nucleic Acids Res*, **47**, D203-D211.
3. Tate, J.G., Bamford, S., Jubb, H.C., Sondka, Z., Beare, D.M., Bindal, N., Boutselakis, H., Cole, C.G., Creatore, C., Dawson, E. *et al.* (2019) COSMIC: the Catalogue Of Somatic Mutations In Cancer. *Nucleic Acids Res*, **47**, D941-D947.
4. Alexandrov, L.B., Nik-Zainal, S., Wedge, D.C., Aparicio, S.A., Behjati, S., Biankin, A.V., Bignell, G.R., Bolli, N., Borg, A., Borresen-Dale, A.L. *et al.* (2013) Signatures of mutational processes in human cancer. *Nature*, **500**, 415-421.
5. Lawrence, M.S., Stojanov, P., Mermel, C.H., Robinson, J.T., Garraway, L.A., Golub, T.R., Meyerson, M., Gabriel, S.B., Lander, E.S. and Getz, G. (2014) Discovery and saturation analysis of cancer genes across 21 tumour types. *Nature*, **505**, 495-501.
6. Landrum, M.J., Chitipiralla, S., Brown, G.R., Chen, C., Gu, B., Hart, J., Hoffman, D., Jang, W., Kaur, K., Liu, C. *et al.* (2020) ClinVar: improvements to accessing data. *Nucleic Acids Res*, **48**, D835-D844.
7. Buniello, A., MacArthur, J.A.L., Cerezo, M., Harris, L.W., Hayhurst, J., Malangone, C., McMahon, A., Morales, J., Mountjoy, E., Sollis, E. *et al.* (2019) The NHGRI-EBI GWAS Catalog of published genome-wide association studies, targeted arrays and summary statistics 2019. *Nucleic Acids Res*, **47**, D1005-D1012.
8. Carithers, L.J. and Moore, H.M. (2015) The Genotype-Tissue Expression (GTEx) Project. *Biopreserv Biobank*, **13**, 307-308.
